# Changes in lower incisor irregularity during treatment with oral sleep apnea appliances

**DOI:** 10.1007/s11325-016-1456-3

**Published:** 2017-01-23

**Authors:** Niclas Norrhem, Hans Nemeczek, Marie Marklund

**Affiliations:** 1 Department of Odontology, Umeå University, SE-901 87 Umeå, Sweden; 2 Centrum för specialisttandvård, ortodonti, Folktandvården Skåne, SE-222 21 Lund, Sweden

**Keywords:** Oral appliances, Mandibular advancement devices, Side effects, Appliance design

## Abstract

**Purpose:**

The purpose of this study is to test the hypothesis that a flexible oral appliance without incisor coverage (OA_Flex_) increases the irregularity of the front teeth compared with a rigid appliance with incisor coverage (OA_Rigid_) in patients treated for obstructive sleep apnea (OSA).

**Method and patients:**

Nineteen patients (10 men) who had used OA_Rigid_ and 22 patients (19 men) who had used OA_Flex_ with a median age of 61 years (IQR of 56 to 67 years) who had been treated during a median period of 2.9 years (IQR of 2.7 to 3.1 years) were included in the study. There was no difference in age (*p* = 0.601) or treatment time (*p* = 0.432) between the two appliance groups. The patients had clinical examinations, responded to a questionnaire, and had impressions taken for plaster casts. The irregularity of the front teeth was measured by Little’s Index, where the combined linear displacement of all the front teeth is assessed. Changes between baseline and follow-up were compared between the two groups.

**Results:**

The OA_Flex_ group increased the irregularity of their lower front teeth by 0.3 mm (*p* = 0.018), while the OA_Rigid_ group had unchanged frontal irregularity (*p* = 0.717). The difference between the groups was significant (*p* = 0.035). There were no changes in the irregularity of the upper front teeth in either group. Patient satisfaction with treatment did not differ between the two appliances.

**Conclusions:**

The present results support the hypothesis that a flexible OA without incisor coverage increases the irregularity of the lower front teeth compared with a rigid OA with incisor coverage.

## Introduction

Side effects are common during the early phases of oral appliance therapy for obstructive sleep apnea (OSA) [1–22]. Some of these adverse effects may result in adherence problems. Most side effects, such as salivation problems and tender teeth or jaws, decrease during the first months of treatment [8]. Bite changes, in contrast, are aggravated by increased treatment time and are therefore the most detrimental side effect [17].

The fixation of the appliance on the teeth with the lower jaw positioned in an advanced position will generate posteriorly directed forces on the upper dentition and anteriorly directed forces on the lower dentition [23]. These forces may result in reduced overjet and overbite and create posterior open bite during longer-term treatment. Studies confirm that this will occur in the majority of the patients [24]. Four studies have assessed changes in space for the teeth or irregular tooth positions [1, 3, 17, 20]. All these four studies used titratable, hard acrylic devices that covered all teeth and did not allow mouth opening. Three of these four studies showed reduced crowding of the lower teeth [3, 17, 20], while one study found no change [1]. One of the four studies observed reduced crowding of the upper teeth [3], while the remaining three studies found no change. The reduction in crowding in the lower arch and not in the upper arch is probably explained by the different force directions that arise from the appliance on the lower and the upper arch, respectively.

The present study was initiated by an observation by a patient at our clinic who had noticed a marked increase in lower incisor irregularity during only a few months’ treatment. There was a marked buccal inclination of a lower incisor, which was verified by comparison with previous plaster casts. At that time, she had been using a fairly new type of oral appliance that is flexible in the lateral dimension and lacks stabilization of the front teeth.

It is generally unknown whether appliance design could influence the degree and type of bite change. Most studies of oral appliances (OAs) have used rigid appliances with full occlusal coverage [1–26]. It is possible that a device that does not cover all the teeth and/or is flexible might cause unexpected bite changes. The aim of this study was therefore to test the hypothesis that a specific brand of OA, which in its original design is both flexible and has no incisor coverage, increased the irregularity of the lower front teeth compared with a rigid OA with frontal coverage.

## Materials and methods

### Study participants

**Fig. 1:**
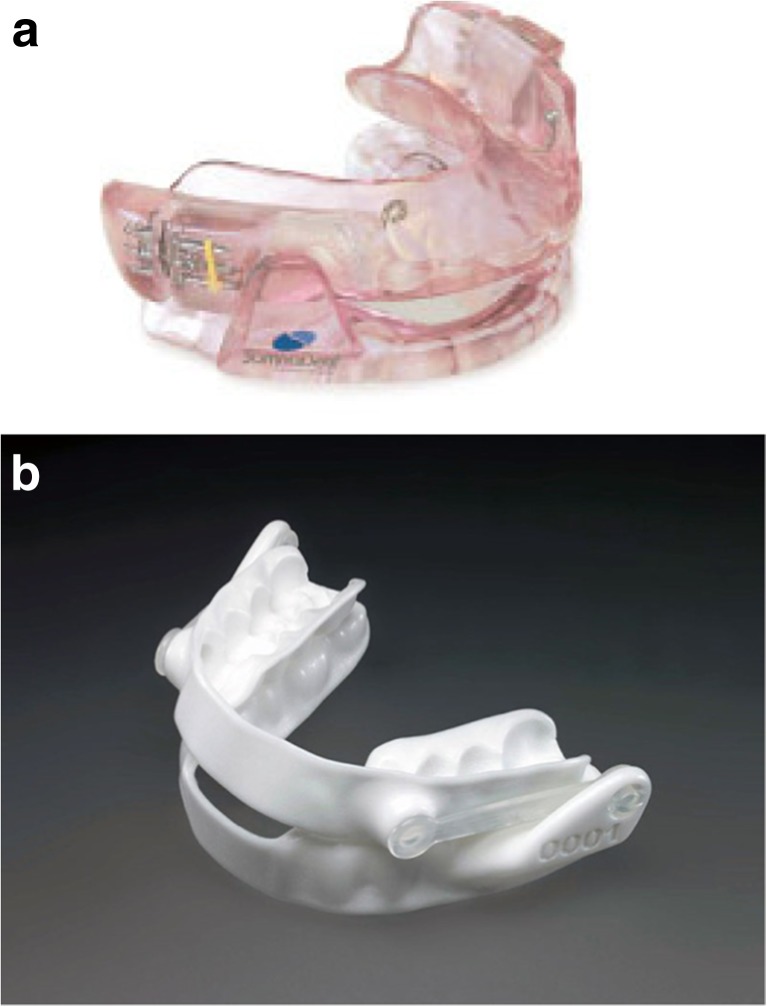
**a** The SomnoDent appliance (OA_Rigid_). **b** The Narval appliance (OA_Flex_)

**Fig. 2:**
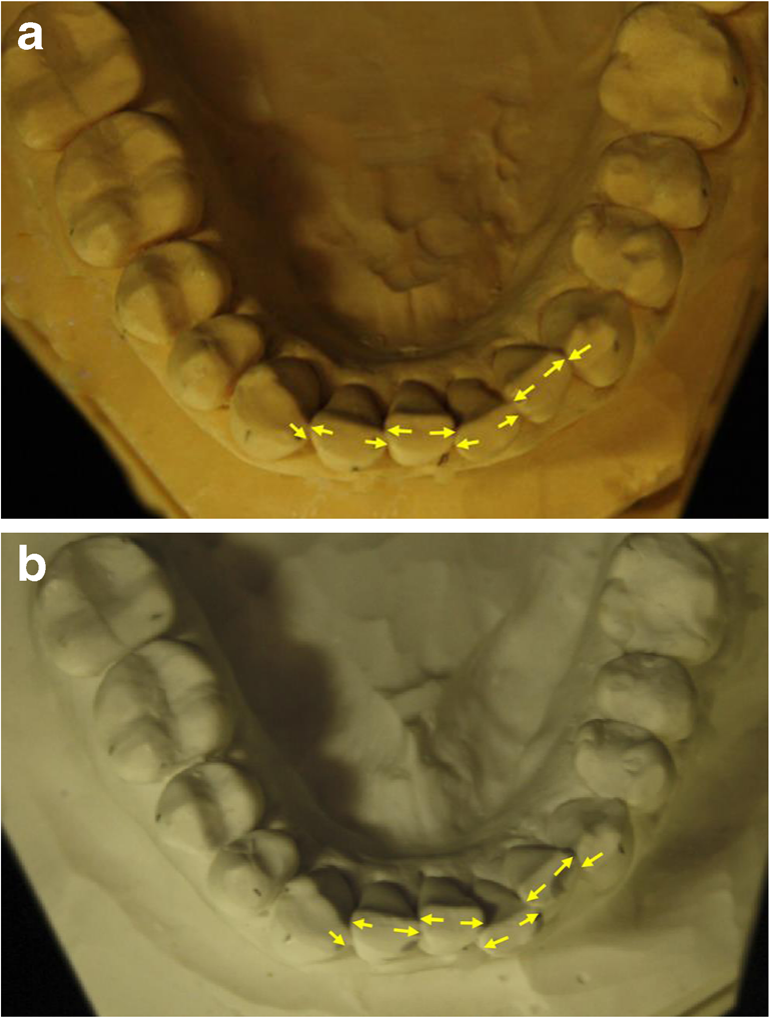
The pictures show photos of the lower jaw of the subject with the greatest increase in irregularity. **a** Before. **b** After. Little’s Irregularity Index was calculated from the summarized distances between the contacts points between two adjacent teeth in the frontal areas. The locations of the measurement points are *marked* in the photos

Consecutive patients who had received either a rigid type of OA with frontal coverage, OA_Rigid_ (SomnoDent, SomnoMed, MAS Nordic, Stockholm) (Fig. 1), or a flexible one, OA_Flex_ without frontal coverage (Narval, ResMed, Lyon, France) (Fig. 2), for the treatment of snoring or obstructive sleep apnea, were considered for inclusion in the study. At the planned 2-year follow-up, patients from each appliance group with as equal treatment periods as possible were selected for a clinical assessment and possible inclusion. The patients had received their appliances from the time we started to use the more flexible type of device in December 2010. The exclusion criteria were inadequate plaster casts (mainly plaster fractures of incisors), adherence for <50% of the nights or less than half of the nights, concomitant use of CPAP, alveolar bone loss on the incisors defined as an attachment level that was located more than 3 mm apical to the cementoenamel junction, or diseases such as dementia that might interfere with the study. The study protocol was approved by the ethics review board at Umeå University, and all the patients gave their written informed consent.

### Clinical assessment

At the follow-up, an extended periodontal status assessment, impressions in alginate for plaster casts, and photographs of the appliances were added to the routine examination of the patients.

### Study cast measurements

All measurements were made blindly with respect to appliance design on the plaster casts at baseline and at follow-up by one investigator (HN).

The irregularity of the front teeth was assessed on the plaster casts using Little’s Irregularity Index [27]. The distances between two contact points or other easily identifiable characteristics on the approximal surfaces of two adjacent teeth were measured. Little’s Index represents the added distances of all front teeth between the mesial surfaces of the canines in one arch. The measurements were made with a measuring microscope (Leitz UWM-Dig-S, Ernst Leitz GmbH, Wetzlar, Germany). The microscope’s accuracy was 0.5 μm. The space between two adjacent teeth was also measured, and all distances between the mesial surfaces of the canines were summarized. The measurements were repeated after a minimum of 1 week, and the mean value was used in the analyses.

Overjet, overbite, and the intercanine distance were measured with an electronic digital sliding caliper to the nearest 0.01 mm.

The degree of mandibular advancement was measured in the premolar area with the device positioned on the baseline plaster casts compared with the plaster casts located in centric occlusion or relation, if applicable, using a transparent sheet with 1 mm squares.

### Questionnaire

The questionnaires were coded and contained questions about the estimated use of the appliances, the subjects’ satisfaction, and side effects. The patients reported the estimated percentage of nights they had used their appliances. They also assessed how often they had used elastics in order to avoid mouth opening on a scale from 0 = “never,” 1 = “sometimes” to 2 = “always.” Satisfaction with the treatment was reported on a scale from 0 = “not satisfied,” 1 = “partially satisfied,” 2 = “sufficiently satisfied” to 3 = “totally satisfied.” Side effects were reported on a scale from 0 = “often,” 1 = “fairly often,” 2 = “seldom” to 3 = “never.”

### Statistical analysis

Wilcoxon’s matched-pairs signed-rank test was performed to test for changes in Little’s Irregularity Index in the frontal areas, frontal spacing, overjet, and overbite. The Mann-Whitney test for independent samples was used to test for differences in baseline characteristics, side effects, and the degree of mandibular advancement between the two appliance groups. Fisher’s exact test was used to test whether there were differences in the proportions of men and women between the appliance groups. IBM SPSS Statistics 24.0 was used for data analysis. A *p* value of less than 0.05 was considered significant.

The sample size was calculated as 15 patients in each group in order to evaluate a change in Little’s Irregularity Index of 1 mm with a power of 0.8 and a significance level of *p* < 0.05.

## Results

### Study population

Of 251 consecutively treated patients, 61 patients had received OA_Rigid_ and 190 patients had received OA_Flex_. Nineteen patients of 24 who had received OA_Rigid_ and 22 patients of 25 who had received OA_Flex_ were selected for a follow-up control, because of similar treatment periods. Five patients from the OA_Rigid_ group were excluded because of alveolar bone loss in the frontal region (1), not used the appliance (1), wanted to wait with the follow-up (2), or used another appliance during the study period (1). Another three patients from the OA_Flex_ group were excluded because of alveolar bone loss in the frontal region (1), not used the appliance (1), or wanted to wait with the follow-up (1). The baseline characteristics did not differ between the two appliance groups (Table 1).

**Table 1:** Baseline characteristics

	OA_Flex_ (*n* = 22)		OA_Rigid_ (*n* = 19)		*p* value
Women/men (% women)	3/19 (14)		9/10 (47)		0.144
	Median	IQR	Median	IQR	
Age (years)	61.65	55.80–66.78	60.80	56.00–66.00	0.601
AHI at start	15.00	10.25–21.00	12.00	6.00–21.50	0.437
Overjet at start (mm)	2.39	1.57–4.15	2.85	2.35–3.34	0.610
Overbite at start (mm)	2.71	1.26–3.98	2.34	1.75–3.48	0.927
Treatment time (years)	2.80	2.61–3.09	3.02	2.68–3.05	0.432
Mandibular advancement (mm)	6.00	4.50–7.00	6.00	4.00–7.00	0.830

### Changes in front teeth irregularity

The irregularity of the lower front teeth increased in the OA_Flex_ group by a median value of 0.30 mm (IQR from 0.00 to 0.69) (*p* = 0.018) and was unchanged in the OA_Rigid_ group, with a median value of 0.00 mm (IQR −0.17–0.19) (*p* = 0.717). There was a significant difference between the appliance groups (*p* = 0.035) (Table 2). Two patients in the OA_Flex_ group and no patient in the OA_Rigid_ group received more than 2 mm change during the study period. There was no change in the irregularity of the upper front teeth (*p* = 0.792).

**Table 2:** Changes by the flexible OA (OA_Flex_) and the rigid OA (OA_Rigid_)

	OA_Flex_ (*n* = 22)		OA_Rigid_ (*n* = 19)		Difference
	Median	IQR	Median	IQR	*p* value
Little’s Index upper (mm)	0.00	−0.21–0.19	0.00	−0.16 –0.09	0.792
Little’s Index lower (mm)	0.30^a^	0.00–0.69	0.00	−0.17–0.19	0.035
Spacing upper (mm)	0.00	0.00–0.00	0.00	0.00–0.00	0.484
Spacing lower (mm)	0.00	0.00–0.00	0.00	0.00–0.00	0.335
Overjet (mm)	0.00	−0.32–0.36	−0.16	−0.27–0.06	0.601
Overbite (mm)	−0.70^b^	−1.22–0.00	−0.36^c^	−0.73 to −0.15	0.266
Intercanine distance upper (mm)	−0.09	−0.20–0.30	−0.17^d^	−0.45–0.07	0.139
Intercanine distance lower (mm)	−0.06	−0.48–0.08	−0.03	−0.17–0.36	0.120

^a^0.018

^b^0.002

^c^0.003

^d^0.036

### Other dental side effects

The intercanine distances showed minor changes (Table 2). The overjet was unchanged, while the overbite decreased (*p* < 0.01) in both groups, with no difference between them (Table 2).

### Subjective effects

The questionnaires regarding satisfaction with and side effects of the OA treatment revealed no significant differences between the two appliance groups (Table 3). The OA_Flex_ group used elastics more frequently than the OA_Rigid_ group (*p* = 0.027).

**Table 3:** Questionnaire regarding effects, side effects, and use at follow-up

	OA_Flex_ (*n* = 22)		OA_Rigid_ (*n* = 19)		Difference
	Median	IQR	Median	IQR	*p* value
Adherence (% of the nights)	95	80–100	90	60–100	0.168
Nightly use (hours)	7	7–7	7	5–7	0.170
Satisfaction with effect on					
Snoring	2	2–3	3	2–3	0.139
Daytime sleepiness	2	2–3	3	2–3	0.145
Overall	3	2–3	3	2–3	0.789
Elastic use	2	2–2	1	0–2	0.027
Side effects	1	0–1	1	0–1	0.649

Satisfaction with the treatment: 0 = “not satisfied,” 1 = “partially satisfied,” 2 = “sufficiently satisfied” to 3 = “totally satisfied.” Elastic use: 0 = “never,” 1 = “sometimes” to 2 = “always.” Side effects: 0 = “often,” 1 = “fairly often,” 2 = “seldom” to 3 = “never”

## Discussion

The present study verified our hypothesis that the flexible OA without frontal coverage, OA_Flex_, produced an increase in the irregularity of the lower front teeth, while the more rigid appliance with full incisor coverage, OA_Rigid_, retained the original appearance of the lower frontal teeth.

A significant increase in the irregularity of the lower incisors of 0.3 mm was found in the present study. This increase was less than expected from our power calculation of 1 mm change. Among the four previous studies that investigate space changes in the incisor region, three report less crowding or irregularities [3, 17, 20]. Rose et al., Chen et al., and Pliska et al. found changes of between −1 to −2 mm in the lower teeth during the 2 and 11 years’ treatment, while Almeida et al. recorded no change after the 4 years’ treatment [1]. All these studies used rigid types of oral appliances with full dental coverage [3, 17, 20]. The decrease in crowding or irregularity of the teeth in these previous studies was probably caused by the forwardly directed forces on the lower jaw with an increase in arch length [1, 20]. An appliance with occlusal and incisor coverage may cause slight flaring of the lower incisors (increased arch length) due to the anteriorly directed forces. With an appliance without a rigid incisor coverage, the posterior teeth also drift forward reducing the arch length due to force direction, but without flaring, and the reduced arch length results in incisor irregularity. The present finding therefore contrasts to previous findings and indicates that an adjustment to the design of the investigated type of flexible OA is advisable.

Two possible design details in this specific brand of oral appliance, the OA_Flex,_ could hypothetically explain the increased irregularity of the lower front teeth after some years’ treatment. Firstly, the lack of support for the lower incisors means that these teeth are free to move in an uncontrolled way compared with what is possible in an appliance that fixes and covers all single front teeth. Secondly, the flexibility of the appliance in the lateral dimension may compress the dental arch and cause incisor irregularities compared with what is possible with a rigid type of device. From the present results, it is impossible to know which if any of these mechanisms caused the detected increase in the irregularity of the lower front teeth.

Our study revealed minor changes in the intercanine distance in both arches. This is in contrast to three previous studies that found an increase in mandibular intercanine distance after 5 to 11 years’ treatment [1, 3, 17]. Rose et al. found no change after 2 years’ treatment. More research is needed to explain differences in space changes for the anterior teeth between appliance designs.

To prevent OA_Flex_ from causing irregularity, full occlusal coverage with contact on all front teeth, as well as the stabilization of the appliance in the lateral dimension, is therefore recommended, based on the present results. These suggested changes are either already available or can easily be achieved in the computerized production process of this appliance in order for it to be more similar to other more rigid designs.

The overjet was unchanged with both appliances in the present study. The overbite changed with median values of −0.7 mm with OA_Flex_ and −0.4 mm for OA_Rigid_, and there was no difference between the groups. Previous studies have shown mean changes in overjet of between −0.2 and −1.5 mm and in overbite of between −0.1 and −1.8 mm after 2 years’ treatment [2, 4, 6, 18, 20, 28]. Consequently, the present results are in line with previous findings.

We had expected an elongation of the incisors resulting in an increased overbite from the OA_Flex_, since this appliance lacks vertical support for the front teeth. It is possible that the contact between the upper and the lower incisors without the appliance during the daytime prevented the incisors from elongation, since the patients had a normal overjet and overbite at the start of treatment. Another explanation might be that the tongue can exert pressure on the incisors during the night, since the appliance lacks material on the lingual side at the front.

Factors such as appliance design, type of bite, and treatment time have been found to influence the degree of change in overjet and overbite during OA treatment. One study revealed no change in overjet and overbite after 4 years’ treatment [18]. That study used a specific OA design, with a lack of buccal coverage on the upper incisors and reinforced lower incisor coverage. Another observational study found fewer changes in overjet and overbite with a soft elastomeric device that covered all the teeth, as well as some parts of the alveolar processes, compared with a hard acrylic one with full occlusal coverage that was mainly fixed to the teeth [11]. A specific orthodontic oral appliance with incorporated forces to counteract the posteriorly directed forces on the upper front teeth showed positive effects on overjet changes compared with a control device in a small group of patients [29]. Consequently, a comparison of side effects between appliance designs has essentially not been made. This lack of knowledge is probably explained by the long treatment time that is needed in order to be able to compare tooth movements between various device designs. More research in this field is therefore needed.

There was no difference in patient satisfaction between the two appliance designs. Most likely, changes in the design of the flexible device to make it more rigid will therefore not influence the subjects’ treatment satisfaction.

Elastic bands were more frequently used with OA_Flex_ than with OA_Rigid_ in our study. One possible explanation may be that elastics usually have to be applied every day to the OA_Rigid_, while they can stay in place until they are worn out on the OA_Flex_. In addition, one study has shown that patients prefer to use elastics on OA_Flex_ [30], while this is unknown for OA_Rigid_. Although unknown, the use of elastics in a flexible device might produce additional unforeseen changes in the dentition.

There are limitations to the present study. First, the study was retrospective, which may have introduced some bias in terms of patient selection. Patients who had experienced bite changes with the appliance might have stopped using it. On the other hand, the results of the study confirmed the complaint from our patient. Complaints of this kind are fairly rare, since most patients are unaware of bite changes. Between 4 and 14% [12, 15] of patients have been reported to notice occlusal changes, although 86% of patients have been found to have these objective changes [1]. After completion of this study, a few more patients have spontaneously reported the same complaint as the patient who was the reason we started this study.

In conclusion, the present results indicate that a flexible type of OA without incisor coverage increases the irregularity of the lower front teeth compared with a more rigid OA with incisor coverage.
